# Sublingual microcirculatory assessment on admission independently predicts the outcome of old intensive care patients suffering from shock

**DOI:** 10.1038/s41598-024-77357-y

**Published:** 2024-10-27

**Authors:** Raphael Romano Bruno, Mara Schemmelmann, Johanna Hornemann, Helene Mathilde Emilie Moecke, Filiz Demirtas, Lina Palici, Radost Marinova, Dominika Kanschik, Stephan Binnebößel, Armin Spomer, Bertrand Guidet, Susannah Leaver, Hans Flaatten, Wojciech Szczeklik, Maciej Mikiewicz, Dylan W. De Lange, Stanislas Quenard, Michael Beil, Malte Kelm, Christian Jung

**Affiliations:** 1 Medical Faculty, Department of Cardiology, Pulmonology and Vascular Medicine, Heinrich-Heine-University Duesseldorf, Duesseldorf, Germany; 2 Equipe: épidémiologie hospitalière qualité et organisation des soins, Sorbonne Universités, UPMC Univ Paris 06, INSERM, UMR_S 1136, Institut Pierre Louis d’Epidémiologie et de Santé Publique, Paris, 75012 France; 3 Assistance Publique-Hôpitaux de Paris, Hôpital Saint-Antoine, service de réanimation médicale, Paris, 75012 France; 4 General Intensive care, St George’s University Hospitals NHS Foundation trust, London, UK; 5 Department of Clinical Medicine, Department of Anaestesia and Intensive Care, University of Bergen, Haukeland University Hospital, Bergen, Norway; 6 Centre for Intensive Care and Perioperative Medicine, Jagiellonian University Medical College, Krakow, Poland; 7 Department of Intensive Care Medicine, University Medical Center, University Utrecht, Utrecht, the Netherlands; 8 General and Medical Intensive Care Units, Hadassah Medical Center, Faculty of Medicine, Hebrew University of Jerusalem, Jerusalem, Israel; 9 CARID (Cardiovascular Research Institute Düsseldorf), Duesseldorf, Germany; 10 Division of Cardiology, Pulmonology, and Vascular Medicine, University Duesseldorf, Moorenstraße 5, 40225 Duesseldorf, Germany

**Keywords:** Microcirculation, Shock, Intensive care, Sidestream-dark field video-microscope, Intravital microscopy, Geriatrics, Medical research, Outcomes research

## Abstract

Shock is a life-threatening condition. This study evaluated if sublingual microcirculatory perfusion on admission is associated with 30-day mortality in older intensive care unit (ICU) shock patients. This trial prospectively recruited ICU patients (≥ 80 years old) with arterial lactate above 2 mmol/L, requiring vasopressors despite adequate fluid resuscitation, regardless of shock cause. All patients received sequential sublingual measurements on ICU admission (± 4 h) and 24 (± 4) hours later. The primary endpoint was 30-day mortality. From September 4th, 2022, to May 30th, 2023, 271 patients were screened, and 44 included. Patients were categorized based on the median percentage of perfused small vessels (sPPV) into those with impaired and sustained microcirculation. 71% of videos were of good or acceptable quality without safety issues. Patients with impaired microcirculation had significantly shorter ICU and hospital stays (*p* = 0.015 and *p* = 0.019) and higher 30-day mortality (90.0% vs. 62.5%, *p* = 0.036). Cox regression confirmed the independent association of impaired microcirculation with 30-day mortality (adjusted hazard ratio 3.245 (95% CI 1.178 to 8.943, *p* = 0.023). Measuring sublingual microcirculation in critically ill older patients with shock on ICU admission is safe, feasible, and provides independent prognostic information about outcomes.

**Trial registration** NCT04169204.

**Supplementary Information:**

The online version contains supplementary material available at 10.1038/s41598-024-77357-y.

## Introduction

Shock is a life-threatening condition characterised by an imbalance between tissue oxygen supply and demand^1^. Microcirculation, comprising vessels < 100 μm in diameter, plays a critical role in tissue perfusion^2^ and homeostasis^3^, making it a central aspect of clinical practice^4^. Despite efforts in intensive care medicine to optimise systemic blood pressure to improve microcirculation, recent studies have shown neutral results^2,5^. Several indirect methods have been proposed to assess microcirculation, such as serum lactate [6], capillary refill time^6^, or the mottling score^7^, which could serve as appropriate resuscitation targets in shock scenarios^8^. Among patients suffering from shock, critically ill very old intensive care patients (VIPs) are generally high-risk patients^9^. Recent VIP studies found a 30-day-mortality between 38.8% and 43%^10–12^. VIPs represent one of the fastest growing subgroups within the intensive care unit (ICU) population^13^. In the European Union, approximately 24.4 million people are expected to be over 85 years of age by 2040. Globally, the proportion of patients aged over 60 years is expected to increase from approximately 12% in 2013 to 21% by 2050^14^. This demographic shift has already had an impact on intensive care unit (ICU) admission rates of VIPs in recent years^15^. Especially in these patients, technologies that might optimize the outcome prediction and treatment optimization in shock, are urgently needed. For this purpose, image-based direct visualisation techniques, including sidestream darkfield-cameras (SDF), have also been developed using intravital microscopy^16^. Numerous studies have shown a correlation between the severity of microcirculatory disturbances and the outcomes in critically ill patients^17–20^. Hand-held devices such as SDF cameras offer a standardised, rapid, and non-invasive assessment of the microcirculation at the sublingual mucosa, the feasibility of which has been positively evaluated even in elderly dehydrated patients^21^. The results may correlate with microcirculation in relevant territories such as the intestinal mucosa, thus serving as a suitable surrogate parameter for whole-body microcirculation^2,22,23^. Automated analysis of video sequences by the AVA 4.3 C software provides an objective evaluation of microcirculatory variables, minimising dependence on subjective visual interpretation^24^. Recently, a large multicentre randomised controlled trial (DAMIS) found no survival benefit from repeated SDF-based treatment optimisation in critically ill shock patients^25–28^. The current study aims at increasing knowledge and create potential cut-offs in old ICU patients for potential use in future clinical trials. Therefore, we conducted this prospective observational study for distinct microcirculatory phenotyping in a particularly vulnerable subgroup of old patients suffering from shock. In sum, this study aimed to test the hypothesis that sublingual microcirculatory assessment on ICU admission predicts 30-day mortality in vulnerable patients aged 80 years or older.

## Methods

### Study design

The Ethics Committee of the University of Duesseldorf, Germany, provided the principal ethical permission. All methods were performed in accordance with the relevant guidelines and regulations. Written informed consent from patients or their surrogates was mandatory. The trial (Very old intensive care patients—perfusion, VIPPER) was registered on clinicaltrials.gov (NCT04169204) on November 14th, 2019.

### Patient selection

To be included, patients had to be 80 years old or older suffering from shock on intensive care unit (ICU) admission. For this study shock was characterised as the requirement for vasopressors despite adequate fluid resuscitation and an elevated lactate level (> 2 mmol/L) indicating of hypotension and hypoperfusion^29^. Determination of adequate fluid replacement was left to the discretion of the attending intensivist, as described previously^25^. Inclusion in the study necessitated the provision of a signed informed consent. When patients could not provide consent, a provisional informed consent form was signed by a medical witness or legal guardian. Exclusion criteria encompassed inaccessibility of the sublingual mucosa, inability to obtain measurements during the inclusion period due to ongoing examinations/treatments/terminal patient status, uncooperative/weakly sedated patients, or infectious conditions that presented a high probability of respiratory viremia, including but not limited to Covid-19 and Influenza. This refinement was necessitated by the stringent guidelines and local standard operating procedures implemented during the initial phase of the Covid-19 pandemic, which mandated strict limitations on non-essential airway interventions and diagnostics to safeguard patient and staff safety and comply with urgent public health directives. Consequently, patients with these infectious conditions were excluded from the VIPPER study to ensure adherence to these critical operational protocols and to maintain the integrity and safety of the study for all participants.

### Sublingual measurement

The assessment of microcirculation in the sublingual region was conducted using the MicroScan USB3 (MS-U) sidestream darkfield (SDF) video-microscope, both upon admission to the Intensive Care Unit (ICU) (within a maximum time frame of 4 h) and 24 h later (± 4 h) following ICU admission. After the measurement, the four highest-quality video sequences, captured at distinct locations on the sublingual mucosa, were evaluated using the AVA 4.3 C algorithm. The resultant variables from these sequences were averaged for analysis^24^. Investigators did not manually calculate any variables. A proof-of-concept study was undertaken to establish the feasibility of this particular method for microcirculation measurement^30^. Subsequently, the quality of the videos was evaluated by trained personnel in a blinded manner, employing the microcirculation-imaging-quality-score (MIQS)^31^.

Furthermore, additional patient-related information was gathered in both cohorts, encompassing macrocirculatory variables such as blood pressure, supplementary microcirculatory parameters such as mottling score or capillary refill time, blood gas analysis values, and administered medications. The microcirculatory assessment was conducted in a blinded manner, independent of the treatment team. A single investigator performed both consecutive measurements. The De Backer Density and the Number of Crossings denote the count of detectable vessels, while the perfused De Backer Density and the Perfused Number of Crossings represent vessels exhibiting visible microcirculatory flow. The Percentage of Perfused Vessels is calculated as the proportion of perfused vessels relative to the total number of all vessels. Small vessels (capillaries) are defined as those smaller than 20 μm^32^.

Patients were retrospectively divided into two risk categories according to their sPPV on admission. The cut-offs were chosen *a posteriori* with an sPPV equal to or greater than 83% as “preserved” and values less than 83% as “impaired” microcirculation based on the included median value of the cohort. There is no commonly accepted definition for cut-offs. Other classifications use 95% as “healthy,” 81–94% as “unhealthy,” and values less than 80% as “dangerous,” depending on the literature from clinical studies^33,34^ and healthy volunteers^35–37^. Other studies consider a PPV < 90% abnormal^16,38^.

### Definition of limitation of life-sustaining therapy

If a patient had a medical indication for specific intensive care measures (typically including intubation and ventilation, cardiopulmonary resuscitation, catecholamine therapy, or haemodialysis), but these interventions were not administered due to the patient’s explicit will, ethical considerations, or in the context of a palliative care situation, such instances were categorised as “withheld therapy.” On the other hand, if any of these measures were initiated but the patient subsequently expressed a desire to terminate the therapy during the course of intensive care, such cases were defined as “withdrawn therapy”^25,39^.

### Cause of shock and comorbidities

Determining the cause of shock was primarily based on aetiology rather than underlying physiology. Specifically, patients with a primary infectious aetiology were classified as experiencing “septic shock” following the Sepsis-3 criteria^29^, and in case of a primary cardiac aetiology (e.g., acute myocardial infarction or acute heart failure) as “cardiogenic shock.” Comorbidities were obtained from health records, and their presence was documented in a binary manner. If a particular condition was identified, irrespective of its severity or aetiology, it was recorded as “present”; otherwise, it was noted as “not present.”

### Statistical analysis

The primary outcome was 30-day mortality. Continuous data points were expressed as median ± interquartile range. Statistical significance in differences between independent groups was calculated using the Mann-Whitney U-test or student’s t-test depending on the data distribution. Categorical data are expressed as numbers (percentages). The Chi^2^ test was applied to test differences between groups for their statistical significance. Univariate and multivariate Cox regression analysis calculated hazard ratios with 95% CI. Multivariate Cox regression was chosen for adjusting patient age, gender and the Sequential Organ Failure Assessment (SOFA) score in recognition of its suitability for analysing time-to-event data, such as 30-day mortality. Patient age served as a demographic variable. Including the SOFA score allowed for considering baseline organ dysfunction severity, enhancing the precision of assessing the impact of other factors on time to the event of interest based on the current evidence^25,40–42^. An additional Cox proportional hazards regression analysis was conducted to evaluate the impact of sPPV on 30-day mortality. Kaplan–Meier curves were depicted to compare the cumulative incidence of mortality after 30 days. The coefficient of variation (cv%) was calculated by dividing the standard deviation by the mean and multiplying the result by 100 to express it as a percentage. Missing values were defined as “missing values,” and a complete-cases analysis was conducted. Unless otherwise stated, there were no missing values for the variables examined in the statistical analysis. Baseline and other variables that are not outcomes were not tested for statistically significant differences. For lactate kinetics, Delta6h and Delta24h were calculated as described previously^40,43^. All tests were two-sided, and a p-value of < 0.05 was considered statistically significant. Analyses were conducted using IBM SPSS Statistics (Version 28). Python 3.12.4 with Visual Studio Code (Version 1.92.0, Microsoft, Redmond, Washington, USA) was used with the lifelines library to compute and display Kaplan-Meier curves, employing the KaplanMeierFitter function for survival analysis and matplotlib for visualizing the results, including formatting the y-axis as percentage values using PercentFormatter. was used for the depiction of Kaplan-Meier comparison.

## Results

### Patients’ baseline characteristics

**Fig. 1:**
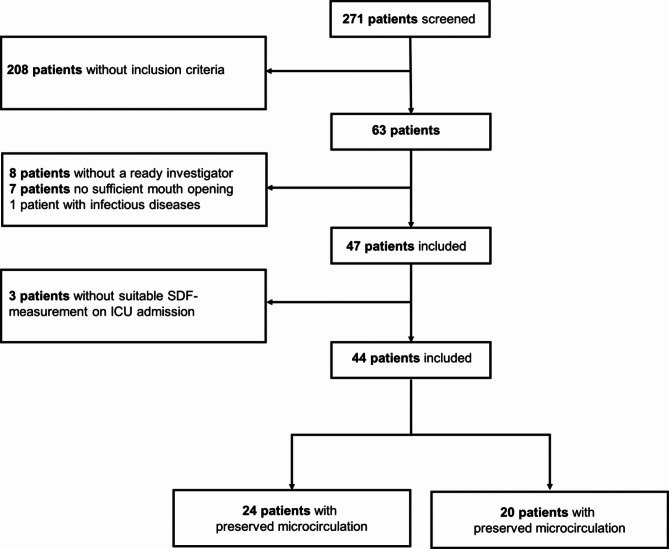
Consort diagram.

From September 4th, 2022, to May 30th, 2023, 63 patients were screened, with 47 meeting the inclusion criteria for shock. Three patients had to be excluded afterward due to missing a suitable SDF measurement on admission (Fig. 1). Thus, 44 patients were analysed and dived according to the median sPPV: 24 patients (55%) demonstrated a preserved, and 20 patients (46%) had an impaired microcirculation. Table 1 provides a detailed comparison between patients with preserved and impaired microcirculation, including various demographic and clinical parameters. A significant difference in gender distribution was observed, with 70% males in the impaired microcirculation group compared to 29% in the preserved microcirculation group (*p* = 0.007). There were no significant differences in age between the two groups, with a mean age of 85 years (± 1) for preserved microcirculation and 84 years (± 1) for impaired microcirculation (*p* = 0.476). Body temperature, respiratory rate, mean arterial pressure, systolic and diastolic arterial pressures, heart rate, SOFA score on admission, body mass index, weight, and length did not differ significantly between the groups. The cause of shock, categorised as sepsis, cardiogenic, neurological, or haemorrhagic, did not exhibit a significant difference (*p* = 0.594). Comorbidities, including heart failure, chronic kidney disease, malignancy, diabetes mellitus, chronic obstructive pulmonary disease, liver cirrhosis, smoking history, and arterial hypertension, also did not show any significant differences between the two groups.

**Table 1:** Baseline characteristics on ICU admission.

	Overall	Preserved microcirculation	Impaired microcirculation	*p*-value
Male gender	21 (48%)	7 (29%)	14 (70%)	**0.007**
Age [years]	84.28 ± 3.85	84.70 ± 0.89	83.68 ± 0.84	0.476
Body temperature [°C]	35.75 ± 1.51	35.92 ± 1.24	35.54 ± 1.79	0.353
Respiratory rate [/Minute]	18.23 ± 6.10	17.79 ± 5.70	18.70 ± 6.60	0.731
Mean arterial pressure [mmHg]	70.06 ± 13.84	71.42 ± 13.82	67.25 ± 14.43	0.337
Systolic arterial pressure [mmHg]	108.38 ± 21.97	109.21 ± 18.43	105.00 ± 25.28	0.540
Diastolic arterial pressure [mmHg]	51.49 ± 12.85	53.08 ± 12.82	49.65 ± 13.48	0.395
Heart rate [/Minute]	86.47 ± 23.06	84.63 ± 20.88	89.95 ± 25.84	0.463
SOFA on admission	9.33 ± 3.66	9.64 ± 3.39	9.38 ± 3.74	0.826
Body mass index	25.80 ± 4.32	26.30 ± 0.82	25.35 ± 1.21	0.556
Norepinephrine [µg/kg/minute]	0.30 ± 0.28	0.27 ± 0.22	0.34 ± 0.34	0.404
Cause of shock				0.594
Sepsis	9 (19%)	4 (17%)	5 (25%)	
Cardiogenic	30 (68%)	16 (67%)	14 (70%)	
Neurological	1 (2%)	1 (4%)	0 (0%)	
Haemorrhagic	4 (9%)	3 (13%)	1 (5%)	
Comorbidities				
Heart failure	12 (26%)	8 (33%)	4 (20%)	0.323
Chronic kidney disease	16 (34%)	11 (46%)	5 (25%)	0.153
Any Malignancy	6 (13%)	2 (8%)	4 (20%)	0.261
Diabetes mellitus	16 (34%)	7 (29%)	9 (45%)	0.277
Chronic obstructive pulmonary disease	9 (19%)	5 (21%)	4 (20%)	0.946
Liver cirrhosis	1 (2%)	1 (4%)	0 (0%)	1.000
Smoking history	7 (16%)	3 (15%)	4 (31%)	0.279
Arterial hypertension	29 (66%)	15 (63%)	14 (70%)	0.601

Significant values are in [bold].

### Video quality

A total of 354 videos underwent retrospective quality control conducted by blinded investigators. Approximately 70% of the videos were deemed good or acceptable based on the quality assessment. The analysis of the most frequent error points, considering all Medical Image Quality Standard (MIQS) criteria, revealed that “stability” accounted for 22%, while “pressure” constituted 33% of the identified issues. The primary reasons for videos being categorised as “not acceptable” included pressure (39%; CV% 247%), stability (24%; CV% 323%), content (23%; CV% 329%), and focus (15%; CV% 420%).

### Microcirculation on ICU admission

The cox-regression of all patients revealed that sPPV was associated significantly with 30-day survial (HR 0.976 (95% CI 0.958–0.993, *p* = 0.006)). This indicates that for percent decrease in sPPV, the risk of 30-day survial decreases by approximately 2.4%. The results presented in Table 2 delineate several haemodynamic and microcirculatory parameters among patients with impaired and preserved microcirculation. Capillary refill time and lactate levels on admission demonstrated no significant differences between the two groups (*p* > 0.05). Regarding Sidestream Dark Field (SDF) measurements at baseline, the number of crossings, De-Backer density, number of crossings (small vessels), and De-Backer density (small vessels) did not exhibit statistically significant differences between the groups (*p* > 0.05). However, the perfused number of crossings, perfused De-Backer density, percentage of perfused vessels, and percentage of perfused small vessels were significantly higher in the preserved microcirculation group compared to the impaired microcirculation group (*p* < 0.05 for all variables). Specifically, the percentage of perfused vessels was 92.9 ± 4.2% in the preserved microcirculation group, significantly higher than the 87.4 ± 4.2% observed in the impaired microcirculation group (*p* = 0.014). Similarly, the percentage of perfused small vessels was markedly higher in the preserved microcirculation group (89.1 ± 5.5%) compared to the impaired microcirculation group (71.4 ± 15.9%), with a highly significant difference (*p* < 0.001). These findings suggest that preserved microcirculation is associated with improved perfusion in overall and small vessels compared to impaired microcirculation. Notably, no adverse events that could be related to the SDF measurement were reported in either group.

**Table 2:** Microcirculatory values on ICU-admission including the SDF-measurement.

	Preserved microcirculation	Impaired microcirculation	*p*-value
Capillary refill time [sec]	3.43 ± 0.43	4.11 ± 0.40	0.185
Lactate on admission [mmol/L]	6.18 ± 0.87	5.93 ± 0.88	0.850
SDF measurement at baseline measurement			
Number of crossings (n/mm)	51.50 ± 11.27	47.38 ± 11.82	0.247
De-Backer-density (n/mm)	10.95 ± 2.40	10.12 ± 2.47	0.268
Number of crossings (small) (n/mm)	20.83 ± 11.74	26.94 ± 11.76	0.094
De-Backer-density (small) (n/mm)	4.43 ± 2.50	5.69 ± 2.51	0.103
Perfused number of crossings (n/mm)	48.22 ± 11.10	39.21 ± 14.05	**0.026**
Perfused De-Backer-density (n/mm)	10.25 ± 2.36	8.33 ± 3.00	**0.026**
Perfused number of crossings (small) (n/mm)	18.16 ± 10.13	19.26 ± 10.16	0.721
Perfused De-Backer-density (small) (n/mm)	3.86 ± 2.15	4.11 ± 2.18	0.708
Percentage of perfused vessels [%]	92.94 ± 4.18	87.39 ± 4.18	**0.014**
Percentage of perfused small vessels [%]	89.12 ± 5.50	71.42 ± 15.94	**< 0.001**

Significant values are in [bold].

### Serum lactate values and kinetics

The maximum lactate value during the first 24 h after ICU admission was higher in the impaired microcirculation group (9.9 mmol/L ± 6.3 compared to 7.3 mmol/L ± 5.7). However, the difference did not reach statistical significance (*p* = 0.084). Similarly, in the case of the maximum lactate value during the first 48 h, patients with an impaired microcirculation exhibited a higher, but statistically not significantly different, mean level at 10.2 mmol/L ± 6.1 compared to those with a preserved microcirculation (7.3 mmol/L ± 5.7, *p* = 0.059). Regarding lactate kinetics, Delta6h, there was no difference. The impaired microcirculation group had a mean of 2.6 mmol/L ± 3.3, while the preserved microcirculation group had a mean of 2.0 mmol/L ± 2.6. The p-value for this comparison was 0.256, indicating no significant difference. Lastly, Delta24h values showed a mean reduction of 5.2 mmol/L ± 3.7 for the impaired microcirculation group and 4.2 mmol/L ± 3.7 for the preserved microcirculation group. Again, the difference was not statistically significant (*p* = 0.177).

### Intensive care treatment and treatment limitations

Numerous differences were observed in the application of ICU-specific interventions (see Table 3). Concerning Renal Replacement Therapy (RRT), the impaired microcirculation group exhibited a markedly higher utilisation rate at 35%, in contrast to the preserved microcirculation group at 8% (*p* = 0.029). Conversely, no significant variance emerged for mechanical ventilation between the two groups, with 71% of cases in the preserved microcirculation group and 75% in the impaired microcirculation group (*p* = 0.757). Extracorporeal Life Support (ECLS) displayed a trend, though statistically insignificant, toward reduced incidence in the impaired microcirculation group (0.0%) compared to the preserved microcirculation group (8%), yielding a p-value of 0.186. In the context of life-sustaining therapy (LST) limitations, several parameters were compared between the impaired microcirculation and preserved microcirculation groups. Concerning the withdrawal of therapy, a higher proportion was observed in the impaired microcirculation group at 26% (9 cases), compared to 19% (4 cases) in the preserved microcirculation group, although this difference did not reach statistical significance (*p* = 0.583). However, the withholding of therapy occurred almost equally in the impaired microcirculation group at 53% (10 cases), and in the preserved microcirculation group (57% (12 cases)), with no significant difference noted (*p* = 0.775). The overall incidence of any limitation on life-sustaining therapy (LST) demonstrated a comparable occurrence between the impaired microcirculation group (80% or 16 cases) and the preserved microcirculation group (79% or 19 cases), with a non-significant p-value of 0.946. Examining the timing of any limitation on LST, the impaired microcirculation group experienced limitations significantly earlier after ICU admission (1.4 days ± 1.8) compared to the preserved microcirculation group (8.0 days ± 22.5), although this difference did not attain statistical significance (*p* = 0.123).

**Table 3:** ICU-treatment and treatment limitations.

	Preserved microcirculation	Impaired microcirculation	*p*-value
ICU-Treatment			
Renal replacement therapy	8% (2)	35% (7)	**0.029**
Mechanical ventilation	71% (17)	75% (15)	0.757
Extracorporeal life support	8% (2)	0% (0)	0.186
Limitation of life sustaining therapy (LST)			
Therapy withdrawn	19% (4)	26% (9)	0.583
Therapy withheld	57% (12)	53% (10)	0.775
Any limitation of LST	79% (19)	80% (16)	0.946
Day of any limitation of LST	8.01 (± 22.47)	1.38 (± 1.78)	0.123

Significant values are in [bold].

### Primary outcome

**Fig. 2:**
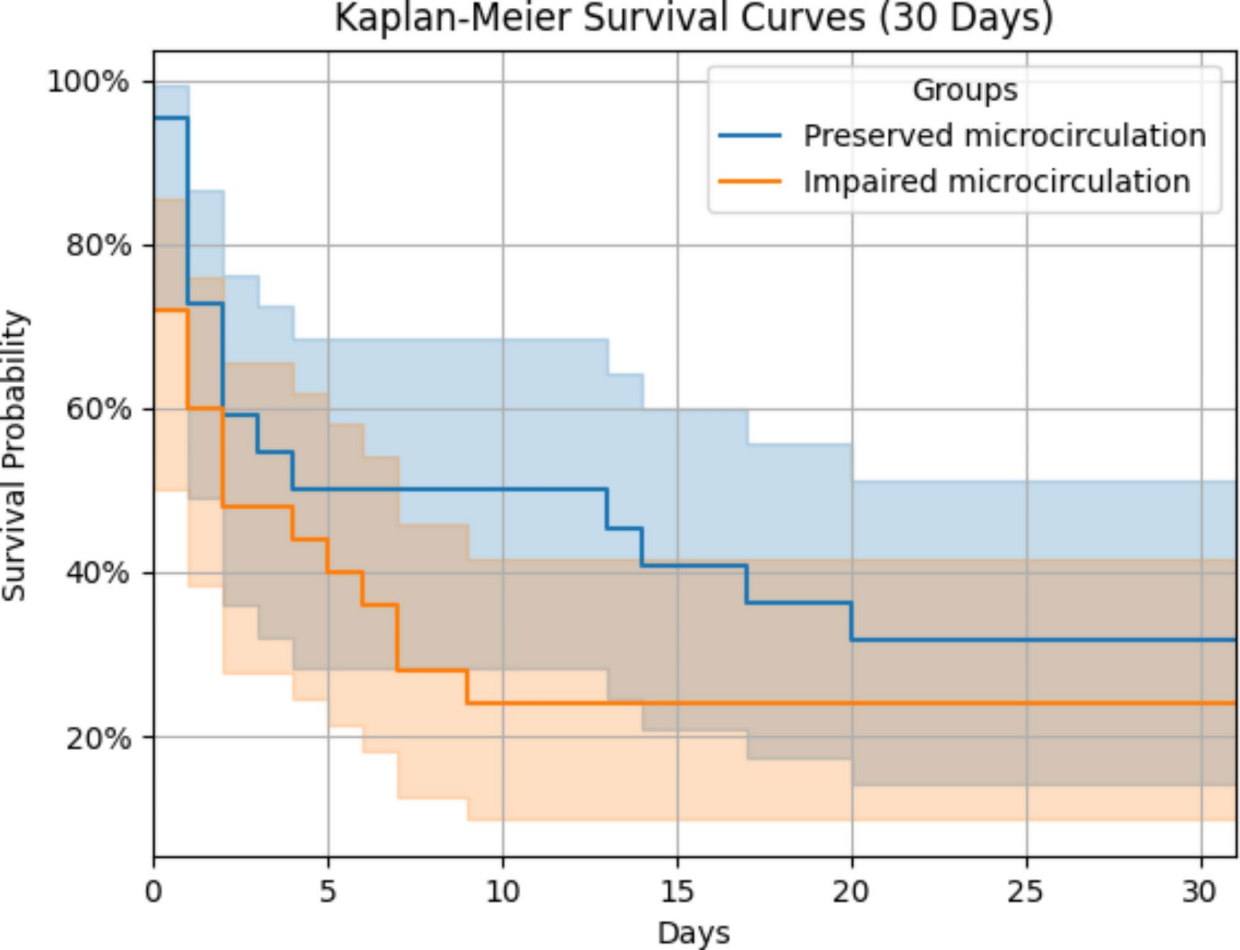
Kaplan-Meier for 30-days probability of survival after ICU-admission with 95% CI. Blue line: Preserved microcirculation; Orange line: Impaired microcirculation. *p* = 0.023 (cox-regression analysis).

Significant differences were observed in patient outcomes between the impaired microcirculation and preserved microcirculation groups (see Table 4). Notably, the length of stay in the ICU was substantially shorter for the impaired microcirculation group (2.6 days ± 3.2) compared to the preserved microcirculation group (7.3 days ± 8.9), with a statistically significant p-value of 0.015. Similarly, the length of hospital stay displayed a significant disparity, with the impaired microcirculation group exhibiting a mean stay of 5.3 days (± 9.7), as opposed to 16.3 days (± 20.9) for the preserved microcirculation group (*p* = 0.019). Examining mortality outcomes, the 30-day mortality rate was notably higher in the impaired microcirculation group, with 90% (18 cases) compared to 63% (15 cases) in the preserved microcirculation group (*p* = 0.036). In the Cox regression analysis, HR for patients suffering from an impaired microcirculation was 2.52 (95.0% confidence interval 1.18 to 5.41, *p* = 0.018, Fig. 2). The association remained significant after adjusting for age, gender and SOFA on admission (adjusted HR 3.245 (95% CI 1.178 to 8.943, *p* = 0.023). ICU survivors evidenced a longer length of stay without reaching statistical significance (7.2 ± 7.3 days versus 3.9 ± 6.8 days, *p* = 0.078). Sublingual microcirculatory values did not differ 24 h after ICU admission (see supplemental Table 1).

**Table 4:** Short- and long-term outcome.

	Preserved microcirculation	Impaired microcirculation	*p*
Length of stay on ICU [days]	7.34 (± 8.90)	2.60 (± 3.21)	**0.015**
Length of stay in hospital [days]	16.26 (± 20.92)	5.30 (± 9.73)	**0.019**
30-days mortality	63% (15)	90% (18)	**0.036**
6-months mortality	75% (18)	90% (17)	0.226

Significant values are in [bold].

### 6 Months follow-up

The 6-month mortality rate, while higher in the impaired microcirculation group at 90% (17 cases) compared to 75% (18 cases) in the preserved microcirculation group, did not reach statistical significance (*p* = 0.226).

## Discussion

The present study was the first to evaluate the microcirculation in a highly vulnerable group of old patients suffering from shock. Patients with an impaired microcirculation on admission to the ICU had a significantly shorter ICU and hospital stay and a significantly higher 30-day mortality, reaching 90% at three months. Almost a quarter of a century ago, De Backer et al.^16^ described critical chances of the sublingual microcirculation in septic and cardiogenic shock^44,45^. In 2020, Bruno et al.^21^ evaluated SDF-measurement as both safe and feasible in 13 older patients suffering from dehydration, but not in shock. In this study, dehydration was associated with a significantly lower percentage of perfused small vessels compared to control (83.1 ± 7.7% versus 88.0 ± 6.0%, *P* < 0.05).

Currently, the most common method of assessing the microcirculation is the measurement of serum lactate and its kinetics. We know from a previous retrospective analysis that both values are associated with the ICU outcome. For example, Bruno et al., included 3299 septic patients, and found that 46% exhibited ≤ 0% lactate clearance, and 54% had > 0% clearance after 6 h. Multilevel logistic regression models were used to examine their impact on intensive care unit (ICU) mortality. Results showed that patients with lactate clearance > 0% had lower sepsis-related organ failure assessment scores and creatinine levels. ICU mortality was significantly lower in this group (14% versus 32%) and remained significant after adjustment (aOR 0.43 95% CI 0.36–0.53; *p* < 0.001)^40^. Similarly, in a subgroup analysis of the COVIP study, a prospective international observation study of patients aged 70 years or older admitted to an ICU due to Covid-19, we analysed 2860 patients. Patients with an elevated baseline serum lactate had a significantly higher ICU- and 3-month mortality (53% vs. 43%, and 71% vs. 57%, respectively; *p* < 0.001). The maximum lactate concentration on day 1 independently predicted ICU, 30-day, and 3-month mortality after adjustment. In patients with baseline lactate ≥ 2 mmol/L, decreasing lactate concentration over time was inversely associated with ICU mortality after multivariate adjustment^43^. Our present study showed slightly higher lactate levels in patients with impaired microcirculation without reaching statistical significance.

In everyday clinical practice, shock resuscitation focuses primarily on normalising serum lactate levels and mean arterial pressure (MAP) with fluids and vasopressors^46,47^. However, there is a constant risk of fluid overload in shock^48^ and it is crucial to recognise that achieving a normal MAP does not guarantee an improvement in microcirculatory flow and tissue perfusion, especially in cases where there is a divergence between macro- and microcirculation^5^. Indeed, in a study of 49 patients, Sakr et al.^49^ demonstrated that those with impaired microcirculation had a significantly worse outcome despite the normalisation of macrohaemodynamic values. Conversely, persistent hyperlactatemia is not always a direct indication of tissue hypoperfusion and should be approached with caution^50^. While high blood lactate levels are correlated with an increased risk of mortality^51,52^, relying on blood lactate alone as a resuscitation target may heighten the risk of “over-resuscitation” and potentially lead to harm^53,54^. In fact, real-time microcirculation measurement has repeatedly been proposed as a relevant clinical tool to assist in titrating fluids and vasopressors more precisely^55,56^. Capillary refill time and serum lactate are established parameters in septic and cardiogenic shock^6,57^. However, the recently published DAMIS trial did not find any benefits for survival using SDF measurement to guide therapy in patients suffering from shock^27^.

By contrast to the microSOAP and DAMIS trials, the present study found that microcirculatory values were significantly associated with outcome. The microSOAP trial (*n* = 501 ICU patients) examined a mixed ICU population and found a significant independent association between lactate levels and several macrohaemodynamic variables with hospital mortality but not microcirculatory variables^33^. On the other hand, the results are in line with the smaller MicroDAIMON study (*n* = 97 ICU patients), which showed an independent association between baseline MFI < 2.6 and the outcome (OR 4.59 95% CI 1.34–15.75, *p* = 0.015)^58^).

However, the aforementioned DAMIS study did not find a significant association between the sPPV on admission and the short-term outcomes. As in the present study, all patients in DAMIS underwent prompt haemodynamic stabilisation before the initial Sidestream Dark Field (SDF) measurement. This circumstance could account for the relatively elevated values of sPPV and the absence of a discernible association between sPPV and the primary study outcome. Microvascular abnormalities exhibit variations across distinct types of shock. Notably, septic shock is characterised by a distinctive microvascular inhomogeneous flow^59^, whereas patients experiencing cardiogenic shock may encounter a reduction in vascular density, with acknowledged heterogeneity also present in cardiogenic shock. Typically, flow-related variables are recommended for risk stratification and management adjustments in fluid therapy rather than those representing capillary density^60^. The microvascular flow index (MFI) is a frequently used flow variable, and an abnormal and low MFI (defined as < 2.6), particularly on the first day of ICU admission, is associated with adverse outcomes^58^. It’s worth noting that MFI necessitates manual interpretation by the investigator, whereas AVA 4.3 C does not offer information about the microcirculatory flow pattern. Alternative approaches, such as the POEM score, emphasise the flow pattern significantly^61^. The study protocol we used for this trial intentionally excluded subjective assessment of the flow pattern by the investigator, as the software lacks the capability to automatically calculate this parameter. The use of an automatic algorithm surpasses individual (subjective) evaluation by providing independent information about microvascular perfusion (yes/no), density, and the percentage of perfused vessels. Earlier studies have identified a satisfactory correlation between a low proportion of perfused vessels (PPV) and low MFI, with a PPV of 88% in the low MFI group and a PPV of 94% in the high MFI group^60^. Furthermore, PPV demonstrates low observer variability^62^.

Notably, the mortality rates from the present study were higher than we expected from the previous studies. However, depending on its aetiology, shock is associated with mortality rates between 30 and 90%^63^. Recently, we used data from the eICU Collaborative Research Database (9,385 patients) to perform a multilevel logistic regression analysis to compare mortality, organ support rates, and length of stay between old (65–79 years) and very old (80 years and older) intensive care patients with sepsis and septic shock. In patients with septic shock, very old patients also showed slightly higher mortality than old patients (38 vs. 36%; aOR 1.50, 95% CI 1.10–2.06; *p* = 0.01)^42^. In another analysis, octogenarians and nonagenarians who were admitted to an ICU showed a mortality of 40 and 45%, respectively after 30 days from ICU admission^9^. Especially old patients who are admitted to an ICU with a higher degree of frailty evidence a significantly higher mortality^12,64^. In VIP-2, there was a relevant difference between ICU- and 30-days mortality (31% versus 42%)^65^.

These associations were independent of the limitations of LST. This is of considerable interest because we know from retrospective studies that any restriction of life-sustaining therapy is associated with significantly increased 30-day mortality and length of stay^66^. Bruno et al.^67^ evaluated 415 acutely admitted ICU patients aged 80 years or more. In this analysis, patients with any limitation of LST had a significantly longer length of stay (144 [IQR 72–293] versus 96 [IQR 47.25–231.5] hours, *p* = 0.026) and an increased 30-day mortality (86% versus 16%, *p* < 0.001).

The present study paves the way for a more in-depth analysis of the implications of real-time microcirculatory assessment in shock resuscitation. Future research efforts should focus on identifying optimal therapeutic interventions tailored to specific patterns of microcirculatory derangement and their relationship with patient-centred outcomes in very old intensive care patients suffering from shock. The VIPPER study clearly underlined that the percentage of perfused small vessels is an early independent indicator for short-term mortality, but to date, without clinical evidence from randomized-controlled trials showing a survival benefit in measuring the sublingual microcirculation, there is no role for routine assessment. To achieve valid data, we need, firstly, to employ technical devices with shorter software processing times and improved video quality. Secondly, larger retrospective analyses are required to generate comprehensive data. Thirdly, we need better data from basic research to determine which parameters should be addressed and how. These findings are necessary for conducting randomized-controlled trials.

### Limitations

Several limitations of this study warrant acknowledgment. Initially, sublingual microcirculation was assessed only at two specific time points. Nonetheless, prior research has demonstrated that evaluating sublingual microcirculation on the first day of ICU admission provides valuable prognostic insights, while subsequent measurements yield limited prognostic information^58^. Conducting measurements upon admission allows for a direct assessment of microcirculation immediately post-resuscitation, revealing potential haemodynamic inconsistencies. Although a minority of videos exhibited lower technical quality, potentially impacting the generalisability of microcirculatory data, it is essential to note that the quality scores obtained align with those reported in other studies^25,68^. Moreover, all videos were conducted by experienced and trained investigators. Additionally, the SDF assessment primarily focused on sPPV, which is considered the most promising and reproducible indicator reflecting a patient’s microcirculatory tissue perfusion. While density values may also be significant, cut-off values are less defined in the literature, and density parameters are predominantly utilised within individuals rather than across individuals. It is crucial to highlight that no established standard approach for optimising microcirculation exists. Furthermore, a considerable percentage of patients exhibited varying degrees of life-sustaining therapy limitations. In 80% of the patients, life-sustaining therapy was either withheld, or withdrawn or both. This is recognised as a notable confounder for short-term mortality in critically ill patients^69^. Lastly, as pointed out by Ospina-Tascón et al.^70^, selecting mortality as the primary endpoint in an intensive care setting may not be the optimal strategy for assessing the value of tools for outcome prediction, especially in studies with smaller number of patients.

## Conclusion

Measuring sublingual microcirculation in critically ill old patients in shock on ICU admission is safe, feasible, and provides additional independent prognostic information about the outcome.

## Electronic supplementary material

Below is the link to the electronic supplementary material.

Supplementary Material 1

Supplementary Material 2

## Data Availability

The anonymised data can be requested from the authors (Dr. Raphael R. Bruno, Raphael.bruno@med.uni-duesseldorf.de) if required.
