# Supplementary material for: Sublingual microcirculatory assessment on admission independently predicts the outcome of old intensive care patients suffering from shock
Source: Sci Rep. 2024 Oct 27;14:25668. doi: 10.1038/s41598-024-77357-y (PMC11514226; doi:10.1038/s41598-024-77357-y)
Supplement: Supplementary file 1 — Supplementary Material 1 [file 41598_2024_77357_MOESM1_ESM.pdf]

Supplemental Table 1: *Microcirculatory values 24 hours after ICU-admission including the SDF-measurement.*

	**Preserved microcirculation**	**Impaired microcirculation**	p
Mottlinge score	0.76 ± 0.90	0.79 ± 1.12	0.80
Capillary refill time [sec]	2.88 ± 1.26	3.43 ± 2.10	0.55
Lactate 24 hours after ICU admission [mmol/L]	2.54 ± 2.32	5.13 ± 5.05	0.12
Lactate 48 hours after ICU admission [mmol/L]	1.56 ± 0.92	1.74 ± 0.91	0.691
**SDF measurement at baseline measurement**			
Number of crossings (n/mm)	52.69 ± 11.17	44.00 ± 14.73	0.10
De-Backer-density (n/mm)	11.21 ± 2.37	9.35 ± 3.13	0.10
Number of crossings (small) (n/mm)	23.47 ± 13.11	16.55 ± 10.38	0.15
De-Backer-density (small) (n/mm)	5.02 ± 2.80	3.52 ± 2.21	0.14
Perfused number of crossings (n/mm)	48.29 ± 12.48	39.38 ± 14.05	0.10
Perfused De-Backer-density (n/mm)	10.27 ± 2.65	8.37 ± 2.99	0.10
Perfused number of crossings (small) (n/mm)	19.83 ± 10.75	13.38 ± 8.80	0.11
Perfused De-Backer-density (small) (n/mm)	4.22 ± 2.29	2.85 ± 1.87	0.11
Percentage of perfused vessels [%]	91.05 ± 5.38	90.01 ± 9.66	0.74
Percentage of perfused small vessels [%]	87.46 ± 7.82	82.51 ± 13.28	0.26
