# Supplementary material for: Sublingual microcirculatory assessment on admission independently predicts the outcome of old intensive care patients suffering from shock
Source: Sci Rep. 2024 Oct 27;14:25668. doi: 10.1038/s41598-024-77357-y (PMC11514226; doi:10.1038/s41598-024-77357-y)
Supplement: Supplementary file 2 — Supplementary Material 2 [file 41598_2024_77357_MOESM2_ESM.pdf]

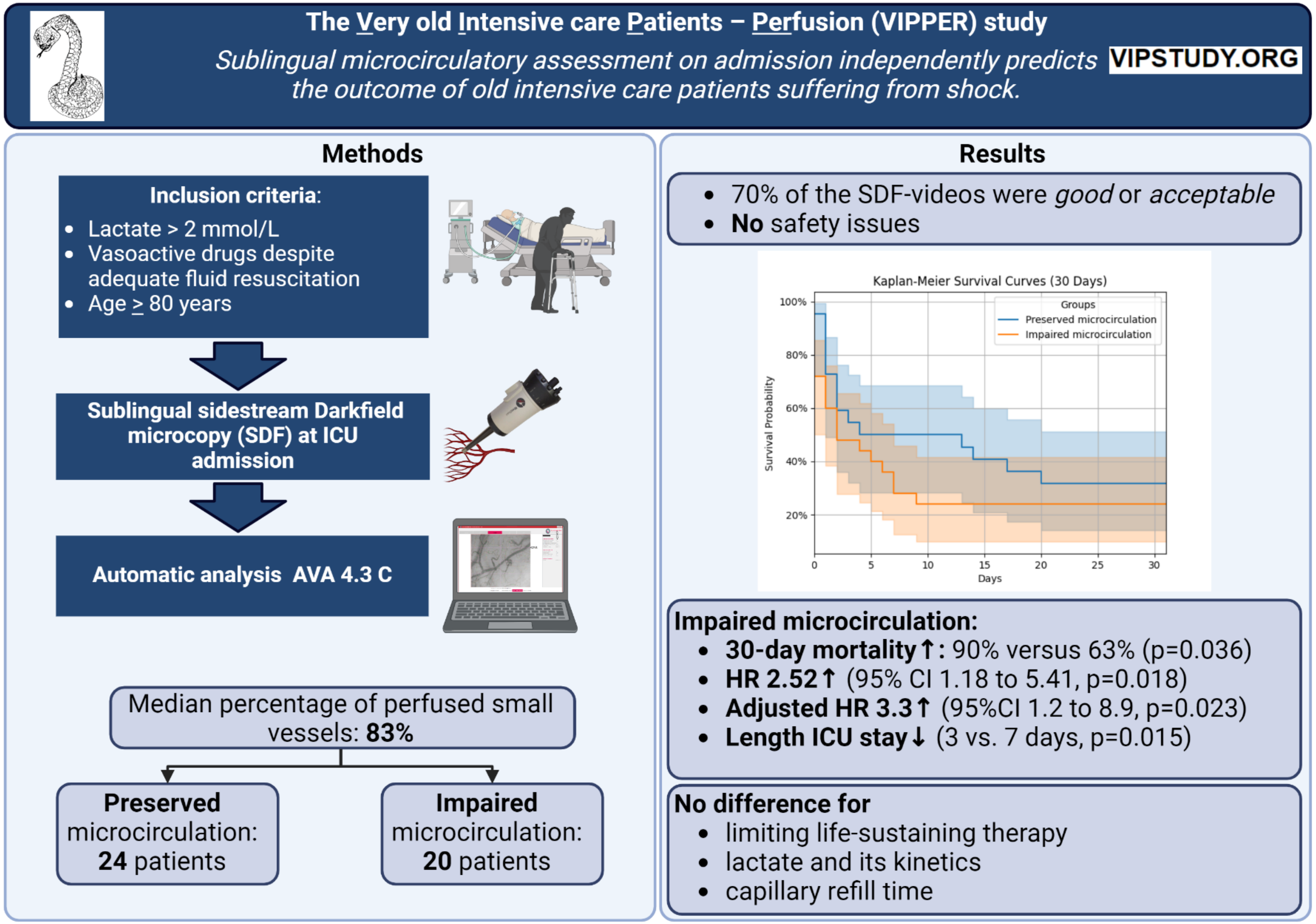

Graphical abstract illustrating the research question, methods, and results of the VIPPER-study. Created with BioRender.com

Supplemental Figure 1: Graphical abstract
